# Convulsive Epidemic of Paris

**Published:** 1830-10-01

**Authors:** 


### LXII.

### Convulsive Epidemic.

At a sitting of the Academy M. Traunoy read a memoir on a convulsive malady, which has reigned for some time epidemically in the commune of Bray-sur-Somme. At the instance of the Prefect, M. Traunoy was summoned to the scene of action, and there found four females affected with the malady. The first was a girl of 17, and her attacks resembled hysteria; they terminated in a deep sleep, and the patient retained no recollection of what had happened. The second uttered cries resembling the crowing of a cock. The third had a kind of hiccup, imitating the noise of a fox. The fourth cut all kinds of capers, leaping like a carp, climbing along a wall with her head downwards, and so forth. M. Traunoy affirms that it is not unusual for the women in the environs of Amiens to utter cries like those of different animals, and even to interrupt divine service in such a manner that they require to be turned out of the church. M. Traunoy alluded to the epidemic *mewing* observed in a convent by Hecquet, which ceased on the physician's declaring that it would be absolutely necessary to bring in a company of soldiers, to flog the fair sisterhood round. The thanks of the Academy were voted to M. Traunoy for his curious paper.

For our parts we have no doubt that the 'epidemic' was nothing less than that mixture of humbug and hysteria, in which the fair sex occasionally delight to indulge. As for the barkers, and pantomimists, and mewers, we protest that M. Hecquet's drum-major and cat-o'-nine tails would prove an infallible specific. If the worthy mayor and M. Traunoy, instead of writing proclamations and memoirs, were to call in the assistance of the arm militant, or souse their patients with some bucketsful of cold water, we have no doubt that the candidates for the 'convulsive epidemic' would speedily vanish. These are the means which succeed à merveille in hospital practice, and although young ladies must be treated more tenderly, yet the principle will hold in all, however prudential considerations may modify the practice.

---

